# Functional intestinal monolayers from organoids derived from human iPS cells for drug discovery research

**DOI:** 10.1186/s13287-024-03685-5

**Published:** 2024-02-29

**Authors:** Tatsuya Inui, Yusei Uraya, Jumpei Yokota, Tomoki Yamashita, Kanae Kawai, Kentaro Okada, Yukiko Ueyama-Toba, Hiroyuki Mizuguchi

**Affiliations:** 1 Laboratory of Biochemistry and Molecular Biology, Graduate School of Pharmaceutical Sciences, Osaka University, 1-6 Yamadaoka, Suita, Osaka 565-0871 Japan; 2 Laboratory of Functional Organoid for Drug Discovery, National Institutes of Biomedical Innovation, Health and Nutrition, Ibaraki, Osaka 567-0085 Japan; 3 Laboratory of Biochemistry and Molecular Biology, School of Pharmaceutical Sciences, Osaka University, Osaka, 565-0871 Japan; 4 Integrated Frontier Research for Medical Science Division, Institute for Open and Transdisciplinary Research Initiatives, Osaka University, Suita, Osaka 565-0871 Japan; 5 Global Center for Medical Engineering and Informatics, Osaka University, Suita, Osaka 565-0871 Japan; 6 Center for Infectious Disease Education and Research, Osaka University, Suita, Osaka 565-0871 Japan

**Keywords:** Intestinal organoids, Small intestine, iPS cell, Drug-metabolizing enzymes, Drug transporters

## Abstract

**Background:**

Human induced pluripotent stem (iPS) cell-derived enterocyte-like cells (ELCs) are expected to be useful for evaluating the intestinal absorption and metabolism of orally administered drugs. However, it is difficult to generate large amounts of ELCs with high quality because they cannot proliferate and be passaged.

**Methods:**

To solve the issue above, we have established intestinal organoids from ELCs generated using our protocol. Furthermore, monolayers were produced from the organoids. We evaluated the usefulness of the monolayers by comparing their functions with those of the original ELCs and the organoids.

**Results:**

We established organoids from ELCs (ELC-org) that could be passaged and maintained for more than a year. When ELC-org were dissociated into single cells and seeded on cell culture inserts (ELC-org-mono), they formed a tight monolayer in 3 days. Both ELC-org and ELC-org-mono were composed exclusively of epithelial cells. Gene expressions of many drug-metabolizing enzymes and drug transporters in ELC-org-mono were enhanced, as compared with those in ELC-org, to a level comparable to those in adult human small intestine. The CYP3A4 activity level in ELC-org-mono was comparable or higher than that in primary cryopreserved human small intestinal cells. ELC-org-mono had the efflux activities of P-gp and BCRP. Importantly, ELC-org-mono maintained high intestinal functions without any negative effects even after long-term culture (for more than a year) or cryopreservation. RNA-seq analysis showed that ELC-org-mono were more mature as intestinal epithelial cells than ELCs or ELC-org.

**Conclusions:**

We have successfully improved the function and convenience of ELCs by utilizing organoid technology.

**Supplementary Information:**

The online version contains supplementary material available at 10.1186/s13287-024-03685-5.

## Introduction

Orally administered drugs are first absorbed, metabolized, and excreted in the small intestine. Since this series of reactions have a significant impact on the pharmacokinetics of drugs, in vitro evaluation of pharmacokinetics in the small intestine is an important consideration in drug discovery research. In current drug discovery research, small intestinal tissues derived from experimental animals and Caco-2 cells, a cell line derived from human colon cancer, are commonly used to evaluate the above responses in vitro. However, there are some problems, such as species differences [1, 2] and significantly lower expression levels of drug-metabolizing enzymes such as cytochrome P450 3A4 (CYP3A4) compared to human small intestinal epithelial cells [3]. To solve these problems, there is a need to establish a culture system that can accurately evaluate pharmacokinetics in the human small intestine [4, 5].

As such an evaluation system, we and others have developed human induced pluripotent stem (iPS) cell-derived enterocyte-like cells (ELCs) [6–14]. With sophisticated differentiation induction protocols, it is becoming possible to generate functional small intestinal epithelial cells from human iPS cells. However, the current protocol requires approximately one month for differentiation into intestinal epithelial cells and does not allow for culture maintenance by passaging after differentiation. Therefore, achieving rapid or large amounts of cell supply is difficult, and the functions of the produced intestinal epithelial cells vary greatly from lot to lot.

To overcome these problems with ELCs, we focused on intestinal organoids, which were originally established from mouse and human intestinal tissues [15, 16]. These organoids are composed of multiple types of intestinal epithelial cells and can be cultured in vitro for long periods. Moreover, they have structural and functional characteristics similar to those of the original tissue. They have also been shown to be useful for pharmacokinetic studies [17–19]. However, the limited supply and the ethical issues surrounding the original tissues have made their use problematic. Therefore, in recent years, several attempts have been made to differentiate intestinal organoids from human iPS cells. These reports sought to establish intestinal organoids not for the purpose of culturing organoids stably, but rather for the purpose of promoting differentiation from intestinal progenitor cells into small intestinal epithelium [10–12]. If intestinal organoids can be established from ELCs and cultured stably, this would be expected to further promote their differentiation into small intestinal epithelium, in addition to shortening the long-term differentiation period, reducing lot-to-lot differences of the differentiated cellular properties, and supplying large amounts of intestinal cells.

In this study, we developed intestinal organoids called ELC-org that can be stably passaged and maintained in culture using human iPS cell-derived ELCs. To be useful for the assessment of intestinal absorption including pharmacokinetics, the culture system must form monolayers. We therefore developed a protocol that successfully produces stable and tight monolayers from ELC-org. The monolayers, which we called ELC-org-mono, not only exhibited CYP3A4 activity comparable to that in primary cryopreserved human small intestinal cells, but also the activities of major drug transporters such as P-glycoprotein (P-gp), breast cancer resistance protein (BCRP) and peptide transporter 1 (PEPT1). Interestingly, the optimization of the medium for monolayer culture was essential for the generation of ELC-org-mono with high drug-metabolizing enzyme expression, while the use of organoid medium was ineffective for monolayer culture. Various analyses were performed to assess the applicability of ELC-org and ELC-org-mono for pharmacokinetics study. The excellent results showed that ELC-org-mono overcome the limitations of the current intestinal models and promise to provide robust and reproducible pharmaceutical assays.

## Materials and methods

### Human iPS cells

The human iPS cell line, YOW and b-iPS cells generated in our previous report were used in this study [20, 21]. The human iPS cell line, Tic, was provided from the JCRB Cell Bank (JCRB: JCRB1331). YOW cells were used mainly for experiments, while other cell lines were used in some experiments to show the robustness of the protocol developed in this study (Additional file 1: Fig. S2). The human iPS cell lines were cultured with StemFit AK02N medium (Ajinomoto) containing 0.1 μg/cm^2^ iMatrix-511 (Nippi).

### In vitro intestinal differentiation

The differentiation protocol for the induction of definitive endoderm (DE) cells and intestinal progenitor cells was based on our previous report with some modifications [9]. Briefly, for the induction of pre-enterocyte-like cells, the intestinal progenitor cells, which were induced for 7 days differentiation, were cultured for 10 days in intestinal final differentiation medium, DMEM high-glucose medium containing 10% KnockOut Serum Replacement (KSR; Thermo Fisher Scientific), 1% non-essential amino acid solution (NEAA; Thermo Fisher Scientific), P/S, 0.5 × B27 supplement minus vitamin A and 1 × GlutaMAX supplement, supplemented with 2 μM SB431542 (SB; FUJIFILM Wako Pure Chemical Industries), 3 nM LY2090314 (LY; MedChem Express), 100 nM 1α,25-dihydroxyvitamin D3 (VD3; Cayman Chemical), and 50 ng/mL epidermal growth factor (EGF; R&D Systems). The pre-enterocyte-like cells were dissociated into single cells and seeded on Matrigel-coated cell culture inserts (24-well plate, 0.4 μm pore size, PET Membrane, Corning) or 96 well plates (Thermo Fisher Scientific) to a density of 5.0 × 10^5^ cells/well. For the induction of enterocyte-like cells, the pre-enterocyte-like cells were cultured for 10 days in intestinal final differentiation medium supplemented with 3 μM PD0325901 (PD; FUJIFILM Wako Pure Chemical Industries). This medium is defined as intestinal maturation medium (IMM) **(**Additional file 1: Table S1**)**. The enterocyte-like cells produced by this process were defined as ELCs.

### Establishment and maintenance of intestinal organoids from ELCs (ELC-org)

Intestinal organoids were established from ELCs. ELCs were scraped and suspended by pipetting with PBS, then resuspended with Matrigel (Corning). Then, 25–40 µL of the organoid suspension was applied to the center of each well of a 24-well plate (Thermo Fisher Scientific). The Matrigel was polymerized for 10 min at 37 °C, and 400 µL/well of an organoid culture medium (OCM) developed in a previous report [22]. For Wnt3a in OCM, we used a conditioned medium derived from HEK293 cells stably expressing mouse Wnt3a (PA-tagged) and human afamin (Target-tagged) [23]. To maintain these organoids, the medium was changed every 2 days and the cells were passaged at 1:4−1:8 every week.

### Proliferative capacity

The number of living cells was evaluated by quantifying intracellular ATP using the CellTiter-Glo 3D Cell Viability Assay (Promega) according to the manufacturer’s instruction. The luminescence intensity was measured with a multimode microplate reader (Berthold Technologies) for 0.25 s per well.

### ELC-org-derived monolayers (ELC-org-mono)

ELC-org-derived monolayers were produced according to our previous report [17]. Briefly, ELC-org were dissociated into single cells and seeded on Matrigel-coated cell culture inserts or 96 well plates to a density of 5.0 × 10^5^ cells/well. The monolayers were cultured for 3–7 days with intestinal maturation medium.

### Primary cryopreserved human small intestinal cells

Primary cryopreserved human small intestinal cells (lot; CHIM6005, ileum of male donors aged 20) were purchased from Discovery Life Sciences. Just after thawing, they were used to measure the CYP3A4, CYP2D6, CYP2C9, and CYP2C19 activities by UPLC-MS/MS analysis.

### qRT-PCR

Total RNA was isolated using ISOGEN (Nippon Gene). As a positive control, total RNA of small intestine was obtained from Total RNA-Human Adult Normal Tissue 5 Donor Pool: Small Intestine (Male donors aged 24 ~ 75, small intestine including duodenum, jejunum, and ileum, Biochain). Using 500 ng of the total RNA, cDNA was synthesized with a Superscript VILO cDNA Synthesis Kit (Thermo Fisher Scientific). qRT-PCR was performed with Fast SYBR Green Master Mix (Thermo Fisher Scientific) using a StepOnePlus Real-Time PCR System (Thermo Fisher Scientific). The Ct values of the target genes were normalized by those of the housekeeping gene, *glyceraldehyde 3-phosphate dehydrogenase* (*GAPDH*). PCR primer sequences are described in Additional file 1: Table S2.

### Transepithelial electrical resistance (TEER) measurement

The TEER values of the ELC-org-mono on cell culture inserts were measured by using Millicell ERS-2 voltohmmeter with STX01 Electrode (MERS00002; Merck). The raw data were converted to Ω × cm^2^ based on the culture insert area. The blank resistance was subtracted from the measured resistance to obtain effective TEER values.

### Transmission electron microscope (TEM) images

Each sample cultured on cell culture inserts (Corning) was fixed in phosphate-buffered 2% glutaraldehyde. Post-fixation, dehydration, embedding, ultrathin sectioning, staining, and observation were performed at the Core Instrumentation Facility, Research Institute for Microbial Diseases, Osaka University.

### Immunostaining

The immunostaining analysis was performed according to our previous report [8]. All antibodies used in this report are described in Additional file 1: Tables S3 and S4.

### Fluorescence-activated cell sorting (FACS) analysis

Single-cell suspensions were fixed with 2% Paraformaldehyde at 4 °C for 10 min, and then incubated with the primary antibody, followed by the secondary antibody. Analysis was performed on a MACSQuant Analyzer (Miltenyi Biotec) and FlowJo software (FlowJo LLC, http://www.flowjo.com/). All the antibodies are listed in Additional file 1: Table S5.

### Luminescent assay for CYP3A4 activity

The measurement of CYP3A4 activity was performed by using P450-Glo CYP3A4 Assay Kit (Promega). Luciferin-IPA was used for the CYP3A4 substrate. The fluorescence activity was measured with a luminometer (Lumat LB 9507; Berthold Technologies) according to the manufacturer’s instructions. The CYP3A4 activities were normalized with the protein content per well by using Pierce BCA Protein Assay Kit (Thermo Fisher Scientific) according to the manufacturer’s instructions. As an inhibitor of CYP3A4, 10 μM ketoconazole (FUJIFILM Wako Pure Chemical) was incubated with the substrate.

### Carboxylesterase 2 (CES2) activity

CES activity was measured based on our previous report [20]. Briefly, cell homogenates were collected and incubated with 10 µM of fluorescein diacetate (FUJIFILM Wako Pure Chemical Industries), a CES2 substrate. As an inhibitor of CES2, 1 mM loperamide (FUJIFILM Wako Pure Chemical Industries) was incubated with the substrate.

### P-gp (MDR1) and BCRP activities

P-gp (multidrug resistance 1; MDR1) activity was measured based on our previous report [24]. Briefly, ELC-org-mono on the cell culture insert were incubated for 90 min with Hank’s Balanced Salt Solution (HBSS; Thermo Fisher Scientific) buffer containing 1 μM ^3^H-labeled digoxin (Digoxin, [3H(G)]-, PerkinElmer) or 10 μM Rhodamine123 (Sigma-Aldrich). The amount of ^3^H-labeled substrates permeated from the apical side to the basal side or from the basal side to the apical side was measured by using a liquid scintillation counter (MicroBeta2; PerkinElmer). Rhodamine123 fluorescent signals were measured with a TriStar LB 941 (Berthold Technologies) using 485 nm excitation and 535 nm emission filters. In the case of BCRP activity, 10 μM ^3^H-labeled estrone sulfate (Estrone 3-sulfate ammonium salt, [6,7-3H(N)]-, ARC) was used as a substrate. As an inhibitor of P-gp and BCRP, 100 μM verapamil (FUJIFILM Wako Pure Chemical Industries) and 100 mM Ko143 (FUJIFILM Wako Pure Chemical Industries) were used, respectively.

### Analysis of ***P***_***app***_

*P*_*app*_ in the transport assay was calculated according to the following equation:

$$P_{app} = \delta {\text{C}}_{{\rm r}} /\delta {\text{t}} \times {\text{V}}_{{\rm r}}/({\text{A}} \times {\text{C}}_{0} )$$

where δC_r_ is the final in the receiver concentration, δt is incubation time, V_r_ is receiver volume, A is the growth area of the cell culture insert, and C_0_ is the initial concentration in the donor compartment.

### Uptake activity of PEPT1

ELC-org monolayers were incubated with HBSS containing 5 μM ^14^C-labeled glycyl-sarcosine (Glycyl-sarcosine, [1-14C]-, ARC) for 90 min at 37 °C. Accumulated glycyl-sarcosine in the monolayer was extracted by adding 70% methanol. Cell lysis solutions were measured by using a liquid scintillation counter (MicroBeta2; PerkinElmer). The PEPT1 activities were normalized with the protein content per well by using a Pierce BCA Protein Assay Kit (Thermo Fisher Scientific) according to the manufacturer’s instructions. As an inhibitor of PEPT1, 100 μM glibenclamide (FUJIFILM Wako Pure Chemical Industries) was used.

### UPLC-MS/MS analysis for the measurement of CYP activities

UPLC-MS/MS analysis was performed to examine the CYP3A4, CYP2D6, CYP2C9, and CYP2C19 activities in ELC-org-mono on the cell culture inserts. The HBSS containing each substrate (Additional file 1: Table S6) was added to the apical chamber in ELC-org-mono. As a positive control, primary cryopreserved human small intestinal cells (just after thawing) were used. After incubation at 37 °C for 60 min, the solution was collected from the apical chamber. UPLC-MS/MS was used to evaluate the production of the following metabolites: 1-hydroxy midazolam (MDOH; a metabolite of CYP3A4), 6β-hydroxy testosterone (TSOH; a metabolite of CYP3A4), 1-hydroxy bufuralol (BFOH; a metabolite of CYP2D6), 4-hydroxy diclofenac (DFOH; a metabolite of CYP2C9) and 4-hydroxy mephenytoin (MPOH; a metabolite of CYP2C19). UPLC analysis was performed using an Acquity UPLC (Waters, Milford, MA) and MS/MS was performed on a Xevo TQ-S (Waters, Milford MA). LC separations were carried out at 40 °C with an Acquity UPLC BEH C18 column, 1.7 μm, 2.1 × 50 mm (Waters). The mobile phase was delivered at a flow rate of 1.0 mL/min using a gradient elution profile consisting of solvent A (0.1% formic acid/distilled water) and solvent B (0.1% formic acid/acetonitrile). Five µL of sample solution was injected into the column. The gradient conditions are as follows; 0 min-2% B, 1.0 min-95% B, 1.25 min-95% B, 1.26 min-2% B, and 1.75 min-2% B. The concentrations of each metabolite were calculated according to each standard followed by normalization to the protein content per well by using Pierce BCA Protein Assay Kit according to the manufacturer’s instructions.

### RNA-seq analysis

Total RNA was isolated using ISOGENE (Nippon Gene). For the evaluation of RNA quality, a BioAnalyzer (Agilent Technologies) and RNA 6000 nanochip (Agilent Technologies) were used, and it was confirmed that all RNA samples had an RNA integrity number (RIN) higher than 7. Library preparation was performed using a TruSeq stranded mRNA sample prep kit (Illumina, San Diego, CA) according to the manufacturer’s instructions. Libraries were converted to libraries for DNBSEQ using an MGIEasy Universal Library Conversion Kit (App-A). Sequencing was performed on a DNBSEQ-G400RS platform (MGI, Shenzhen, China) in 2 × 100 bp paired-end mode. RNA-Seq data were processed using Trimmomatic to trim low-quality bases and Illumina sequencing adapters from the 3′ end of the reads. Reads were mapped to GRCh38.p13 of the human genome using HISAT2 [25]. HISAT2 outputs were fed into featureCounts [26] for transcript quantification. DESeq2 [27] was used to product fragments per kilobase of exon model per million reads mapped (FPKM) values. The integrated Differential Expression and Pathway analysis (iDEP 96) platform [28] (http://bioinformatics.sdstate.edu/idep96/) was used to create the heatmap and perform k-means clustering and GO enrichment analysis. In iDEP.96, DESeq2 was used to identify differentially expressed genes (DEGs).

### Ethical statement

The ethics committees of Osaka University approved this study. All experiments were performed in accordance with the ethical standards of the institutional and national research committees, and with the Declaration of Helsinki.

### Statistical analysis

Statistical analysis was performed using the unpaired two-tailed Student’s t-test or one-way ANOVA followed by Tukey’s post-hoc tests. A value of *p* < 0.05 was considered statistically significant.

## Results

### Establishment of intestinal organoids using human iPS cell-derived small intestinal epithelial cells

**Fig. 1:**
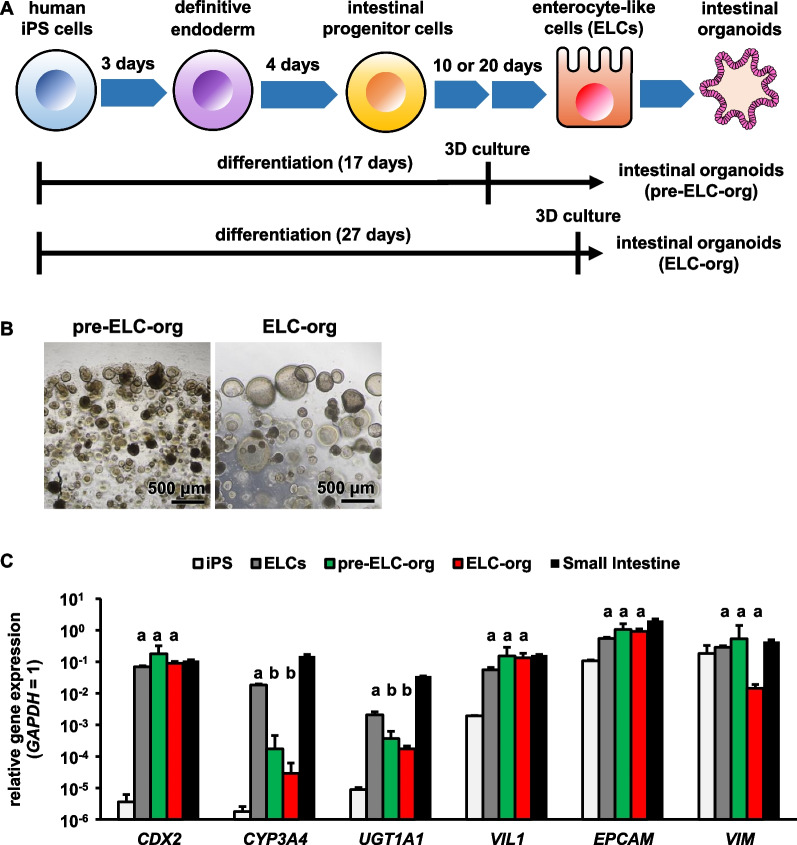
Establishment of pre-ELC-org and ELC-org. (**A**) Diagram of the protocol for generating pre-ELC-org and ELC-org from human iPS cells. (**B**) Phase-contrast images of pre-ELC-org and EC-org are shown. (**C**) The gene expression levels of drug-metabolizing enzymes (*CYP3A4*, *UGT1A1*), intestinal markers (*CDX2*, *VIL*, *EPCAM*) and a mesenchymal cell marker (*VIM*) were examined in human iPS cells, ELCs, pre-ELC-org, ELC-org, and the human small intestine by qRT-PCR. The *GAPDH* expression level was taken as 1.0. Data represent the mean ± S.D. (*n* = 3, biological replicates). Statistical significance was evaluated by one-way ANOVA followed by Tukey’s post hoc test. Groups that do not share the same letter had significantly different results (*p* < 0.05)

Human iPS cell-derived enterocyte-like cells (ELCs) were generated as described in the Materials and Methods section. Since most of the reported organoids were established from human iPS cell-derived hindgut endoderm cells or intestinal progenitor cells [10, 11, 29–32], we first examined an optimal period to establish intestinal organoids from human iPS-derived cells. We generated intestinal organoids during two differentiation induction periods which we defined as pre-ELC-org (days 0–17) and ELC-org (days 0–27) (Fig. 1A). Each organoid could be cultured for over 1 year. Phase contrast microscopic images revealed that the size of ELC-org was larger than that of pre-ELC-org (Fig. 1B). To compare the gene expression levels in each organoid, qRT-PCR analysis was performed. The results showed that there was no significant difference in the gene expression levels of the hindgut differentiation marker (*caudal type homeobox 2*; CDX2), absorbed epithelial cell marker (*villin*; VIL1), and epithelial cell marker (*epithelial cell adhesion molecule*; EpCAM) among ELCs, pre-ELC-org and ELC-org. However, the gene expression levels of the drug-metabolizing enzymes (*cytochrome P450 3A4*; CYP3A4, and *UDP-glycosyltransferase 1A1*; UGT1A1) in both pre-ELC-org and ELC-org significantly dropped as compared with those in ELCs. The gene expression level of the mesenchymal cell markers (*vimentin*; VIM) in ELC-org was lower than that in pre-ELC-org (Fig. 1C). The proliferative capacity of ELC-org was examined (Additional file 1: Fig. S1). pre-ELC-org and ELC-org grew about 500- and 1200-fold in 21 days, respectively. Since the ELC-org grew faster and seemed to have less contamination of mesenchymal cells, ELC-org were used in the following studies. ELC-org could also be generated from ELCs differentiated from other iPS cell lines by the same method (Additional file 1: Fig. S2A). The gene expression profiles in each ELC-org showed a similar trend among the lines (Additional file 1: Fig. S2B).

### Highly functional ELC-org-mono can be produced with the optimal culture medium

**Fig. 2:**
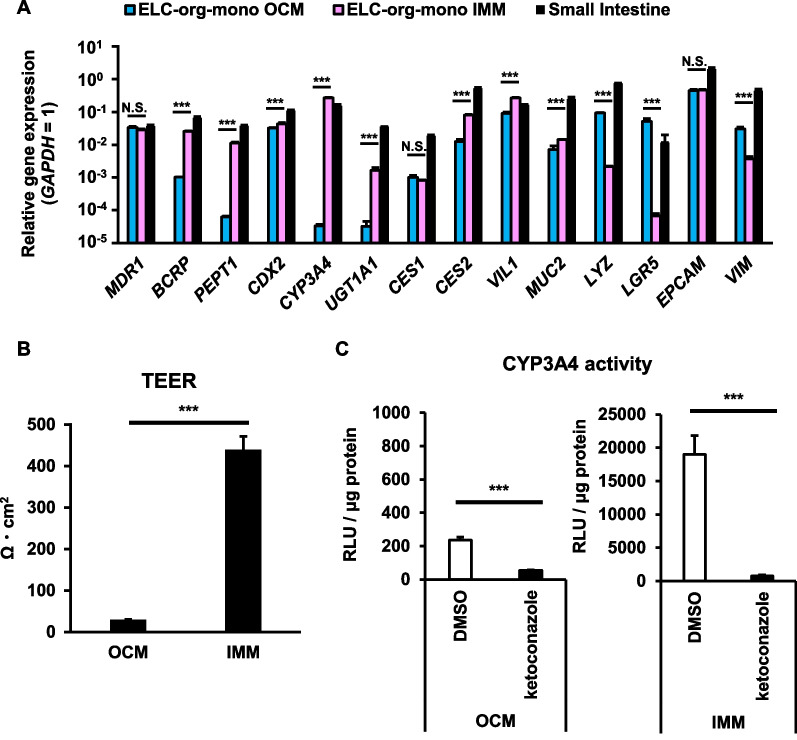
Optimal medium for monolayer culture of ELC-org (ELC-org-mono). (**A**) The gene expression levels of drug transporters (*MDR1*, *BCRP*, *PEPT1*), drug-metabolizing enzymes (*CYP3A4*, *UGT1A1*, *CES1*, *CES2*), intestinal cell markers (*CDX2*, *VIL*, *MUC2*, *LYZ*, *LGR5*, *EPCAM*) and a mesenchymal cell marker (*VIM*) were examined in ELC-org and ELC-org-mono cultured with different media and in the human small intestine by qRT-PCR. The organoid culture medium (OCM) was the same as the medium previously used for the organoid culture, while intestinal maturation medium (IMM) was the same as the final medium previously used for the differentiation of ELCs differentiation from human iPS cells. Details are shown in the Materials and Methods section. The *GAPDH* expression level was taken as 1.0. (**B**) The TEER values in each cell monolayer cultured on cell culture inserts were measured by Millicell-ERS2. (**C**) The CYP3A4 activity in each cell monolayer was examined by using the P450-Glo CYP3A4 assay kit in the presence or absence of 10 µM ketoconazole (a CYP3A4 inhibitor). All data represent the mean ± S.D. (*n* = 3, biological replicates). Statistical analyses were performed using the unpaired two-tailed Student’s t-test (***p < 0.005). N.S. means “Not Significant”

For application to intestinal absorption including pharmacokinetic evaluation, intestinal organoids must be cultured in two dimensions and form tight junctions. We previously showed that human biopsy-derived intestinal organoids were cultured in monolayers by dissociation and seeding as single cells, and that the expression level of CYP3A4 in the cells was increased more than 100-fold by cultivation in two dimensions with organoid culture medium [17]. Therefore, we expected that the gene expression level of CYP3A4, which dropped in ELC-org, would be increased to the comparable level to that in the human small intestine when ELC-org were monolayer-cultured (ELC-org-mono) with the organoid culture medium (OCM). However, this was not the case (Fig. 2A). Interestingly, we found that the gene expression levels of CYP3A4 and UGT1A1 were dramatically enhanced when ELC-org-mono were cultured with the final medium (intestinal maturation medium: IMM) for the differentiation of ELCs (Fig. 2A). In particularly, the gene expression level of CYP3A4 became similar to that in the human small intestine. This result showed that although ELC-org temporarily lost some of the important intestinal functions of the original ELCs by forming organoids, they acquired the same or better functionalities as the original ELCs by forming monolayer, together with use of the optimal culture medium. In addition to the increased gene expression levels of CYP3A4 and UGT1A1, those of some drug transporters, such as breast cancer resistance protein (BCRP) and peptide transporter 1 (PEPT1), were also greatly enhanced by the monolayer culture with IMM. The TEER and CYP3A4 activity were improved when ELC-org-mono were cultured with IMM **(**Fig. 2B and C). These indicates that not only can ELCs be maintained in passaging culture by forming organoids, but also the monolayers with higher functionality can be fabricated. Therefore, in the following studies, ELC-org-mono were cultured with IMM.

Importantly, the gene expression pattern in ELC-org-mono cultured with IMM did not change markedly even after ELC-org were successively passaged (up to 53 times; for more than a year) (Additional file 1: Fig. S4A). This suggests that ELC-org are genetically stable culture systems even after long-term culture.

### Consideration of the optimal monolayer culture period

To determine the optimal culture period for evaluations of drug metabolic and drug transport activities, several assays were performed at different culture periods using ELC-org-mono prepared on the same day. First, the expression levels of major genes related to intestinal function and differentiation were examined by qRT-PCR. The results showed that the expression levels of many genes increased with longer culture periods, but only slightly (Additional file 1: Fig. S5A). The TEER value was high enough to use for pharmacokinetic studies at any culture period (Additional file 1: Fig. S5B). The CYP3A4 activity tended to be higher in the longer monolayer culture period but was sufficiently high even at day 3 (Additional file 1: Fig. S5C). The P-gp activity using Rhodamine123 as a substrate was higher in the early stage (day 3–5) than in the late stage (day 7) (Additional file 1: Fig. S5D). In the following studies, we used ELC-org-mono at 7 days of monolayer culture to assess drug-metabolizing enzyme activities and those at 3 days of monolayer culture to assess drug transport activities.

### Established organoids structurally mimic the small intestine

**Fig. 3:**
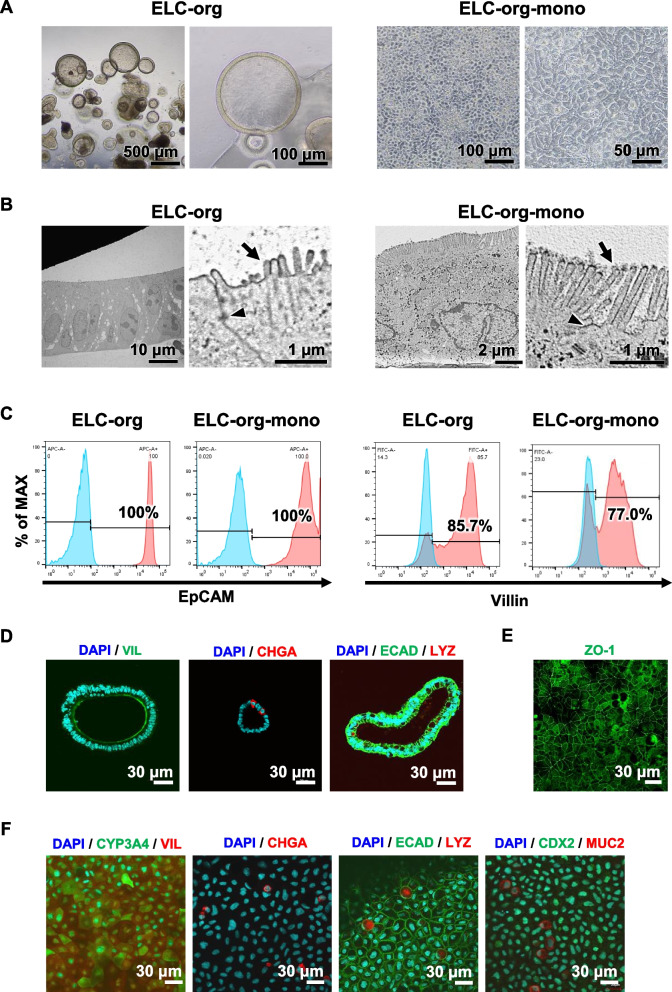
Differences in morphology and protein expression between ELC-org and ELC-org-mono. (**A**) Phase-contrast images of ELC-org and ELC-org-mono are shown. (**B**) TEM images of ELC-org and ELC-org-mono are shown. Microvilli (black arrows) and tight junctions (black arrowheads) are indicated. (**C**) Percentages of EpCAM- and Villin-positive cells in ELC-org and ELC-org-mono were measured by FACS analysis. Negative control (blue) and stained cells (red) are represented. (**D**) The protein expressions of VIL, CHGA, ECAD and LYZ in ELC-org were characterized by immunostaining. (**E**) The protein expression of ZO-1 in ELC-org-mono was characterized by immunostaining. (**F**) The protein expressions of CYP3A4, VIL, CHGA, ECAD, LYZ, CDX2 and MUC2 in ELC-org-mono were characterized by immunostaining

We examined various properties of ELC-org and ELC-org-mono as an in vitro human small intestinal model. First, morphological evaluation was performed **(**Fig. 3A and B). Phase contrast microscopic images revealed a spherical morphology with a smooth surface and a thin cell layer (approximately 10 µm) in ELC-org. ELC-org-mono had columnar morphology. TEM revealed that the cell layer of ELC-org and ELC-org-mono each consisted of a single cell layer, and that microvilli **(**Fig. 3B, right panel arrow) and tight junction formation (Fig. 3B, right panel arrowhead) were formed. The villi were further distended from the surface in the ELC-org-mono. These results suggest that ELC-org became structurally mature when formed into a monolayer.

Next, ELC-org and ELC-org-mono were examined in various ways for expression at the protein level. To confirm the epithelial cell population, FACS analysis was performed targeting EpCAM, an epithelial cell marker, and Villin, an absorptive epithelial cell marker. The results showed that ELC-org had a 100% EpCAM-positive cell rate and a Villin-positive cell rate exceeding 85% (Fig. 3C). This confirmed that ELC-org do not require selection for epithelial cells when used in various pharmacokinetic assays. The positive cell rate for each marker was checked in ELC-org-mono. The results showed that ELC-org-mono also had a 100% EpCAM-positive cell rate, while the Villin-positive cell rate was 77%, lower than that in ELC-org. These results suggest that both ELC-org and ELC-org-mono are composed exclusively of epithelial cells, with the majority being absorptive epithelial cells.

Immunostaining was also performed to confirm the presence of differentiated cells in the human small intestine. It was confirmed that ELC-org contained absorptive epithelial cells (VIL), endocrine cells (CHGA), and Paneth cells (LYZ) (Fig. 3D). The expression of the tight junction marker ZO-1 was confirmed in ELC-org-mono (Fig. 3E). This supported the result of TEM observations. In ELC-org-mono, the presence of goblet cells (MUC2) was also confirmed in addition to the differentiated cells identified in ELC-org (Fig. 3F). These facts suggested that ELC-org-mono are a culture system containing epithelial cells and differentiated cells in the human small intestine.

### ELC-org-mono were universally applicable to pharmacokinetic studies

**Fig. 4:**
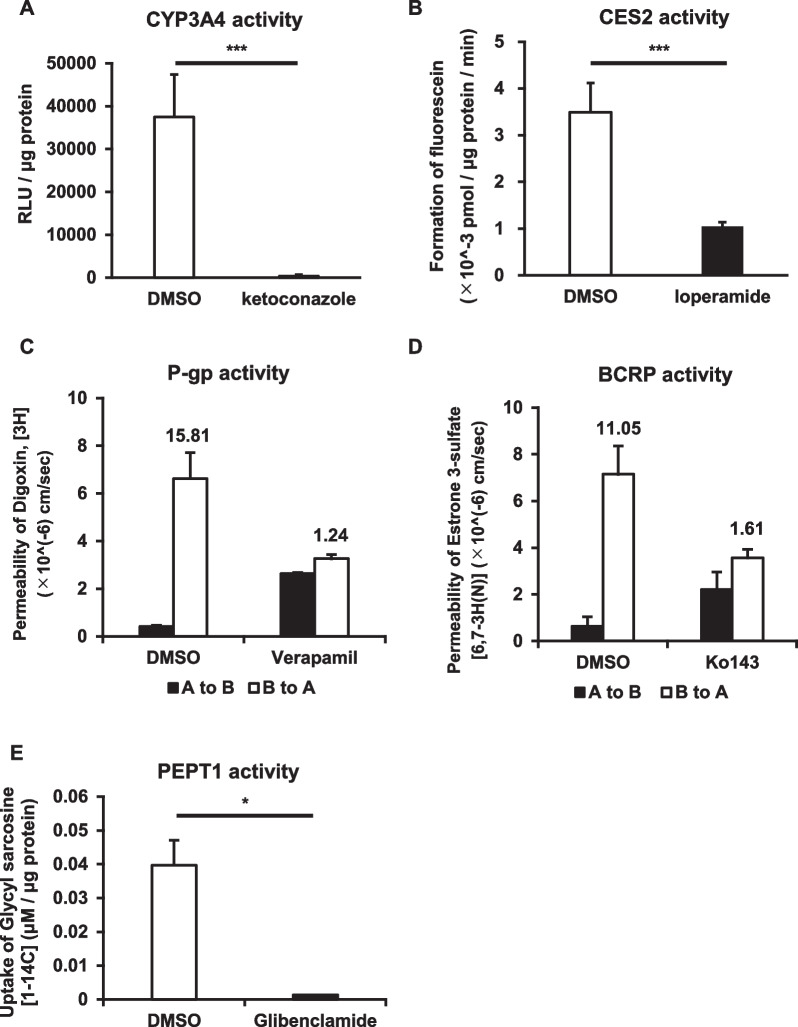
Drug-metabolizing activities and drug transport activities were examined in ELC-org-mono. The CYP3A4 (**A**) and CES2 (**B**) activities were measured. (**C**) The permeabilities of ^3^H-labeled digoxin (substrate for P-gp) in ELC-org-mono with or without 100 µM verapamil (P-gp inhibitor) were measured. The efflux ratio (P_app B to A_/P_app A to B_) of each group is shown above the bar. (**D**) The permeabilities of ^3^H-labeled estrone sulfate (substrate for BCRP) with or without 100 µM Ko143 (BCRP inhibitor) were measured. The efflux ratio (P_app B to A_/P_app A to B_) of each group is shown above the bar. (**E**) The PEPT1 activity was measured by evaluating the amount of ^14^C-labeled Glycyl sarcosine (substrate for PEPT1) uptake into the cells with or without 100 µM glibenclamide (PEPT1 inhibitor). All data represent the mean ± S.D. (*n* = 3, biological replicates). Statistical analyses were performed using the unpaired two-tailed Student’s t-test (*p < 0.05, ***p < 0.005)

To confirm the pharmacokinetic functions of ELC-org-mono, we evaluated these culture systems in various ways. We found that clear drug-metabolizing activities (CYP3A4 and CES2 activities (Fig. 4A and B) and drug transport activities (P-gp, BCRP, and PEPT1 activities (Fig. 4C, D and E) were present, and that each activity was inhibited by inhibitors. Furthermore, the CYP3A4 and P-gp activities in ELC-org-mono did not change markedly even after ELC-org were successively passaged more than 50 times (Additional file 1: Fig. S4B and S4C). These results suggest that the ELC-org-mono have sufficient functionality to be used for drug metabolism and drug transport studies.

The CYP3A4 induction is a major mechanism causing drug-drug interactions. We examined whether ELC-org-mono retain the induction capacity of CYP3A4. Since the intestinal maturation medium (IMM) used for the monolayer culture already contained VD3, an inducer of CYP3A4 [33, 34], ELC-org-mono were cultured in IMM without VD3 (-V) for 5 days, and they were cultured in IMM or IMM without VD3 with/without rifampicin (RIF) (IMM/-V, + RIF/-RIF) for 2 days (Additional file 1: Fig. S6A). The gene expression levels of CYP3A4 in ELC-org-mono were clearly up-regulated by RIF (Additional file 1: Fig. S6B). Note that even when ELC-org-mono were cultured with IMM, which contained VD3, for only 5–7 days, the gene expression levels of CYP3A4 were close to those in the human small intestine and up-regulated by RIF. The gene expression levels of MDR1 and UGT1A1 were also found to be induced by RIF, but the expression levels of other genes were largely unaffected by VD3 and RIF **(**Additional file 1: Fig. S6C). These results suggest that ELC-org-mono retain the induction capacity of CYP3A4.

Next, we examined whether ELC-org can form functional monolayers from cryopreserved cell suspensions. For this purpose, ELC-org were dissociated into single cells and cryopreserved for 2 weeks. We examined the functions of ELC-org-mono produced by seeding them directly onto cell culture inserts. These monolayers were defined as “F-ELC-org-mono”. qRT-PCR analysis showed no negative effects of cryopreservation on gene expression profiles of F-ELC-org-mono (Additional file 1: Fig. S7A). Furthermore, F-ELC-org-mono exhibited sufficient levels of CYP3A4 and P-gp activities (Additional file 1: Fig. S7B and S7C), which were comparable to those in ELC-org-mono without cryopreservation (Fig. 4A and Additional file 1: Fig. S5D).

**Fig. 5:**
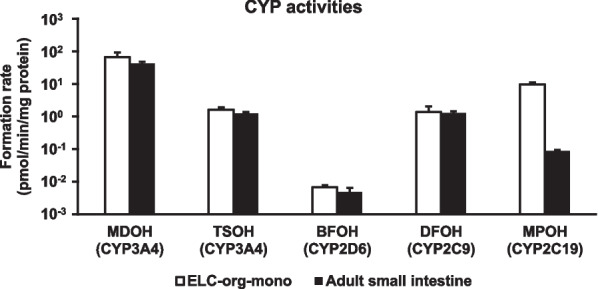
Comparison of drug-metabolizing activities of various CYPs between ELC-org-mono and primary cryopreserved human small intestinal cells. Drug-metabolizing activities of CYP3A4, CYP2D6, CYP2C9, and CYP2C19 between ELC-org-mono and primary cryopreserved human small intestinal cells were examined by quantifying the metabolites of each substrate (MDOH, TSOH, BFOH, DFOH and MPOH) by UPLC-MS/MS analysis. In the case of CYP3A4, midazolam and testosterone were used as substrates. Drug-metabolizing activities in primary cryopreserved human small intestinal cells were measured just after the cells were thawed. All data represent the mean ± S.D. (n = 3, biological replicates)

We also compared the drug-metabolizing activities of various CYPs in ELC-org-mono with those in primary cryopreserved human small intestinal cells (just after thawing) by LC–MS/MS (Fig. 5). The results showed that the activities of CYP3A4, CYP2D6, and CYP2C9 in ELC-org-mono were comparable to those in primary cryopreserved human small intestinal cells. The CYP2C19 activity in ELC-org-mono was higher than that in primary cryopreserved human small intestinal cells. This suggests that ELC-org-mono mimic the human small intestine in their drug-metabolizing capacity.

### RNA-seq analysis of ELCs, ELC-org and ELC-org-mono

**Fig. 6:**
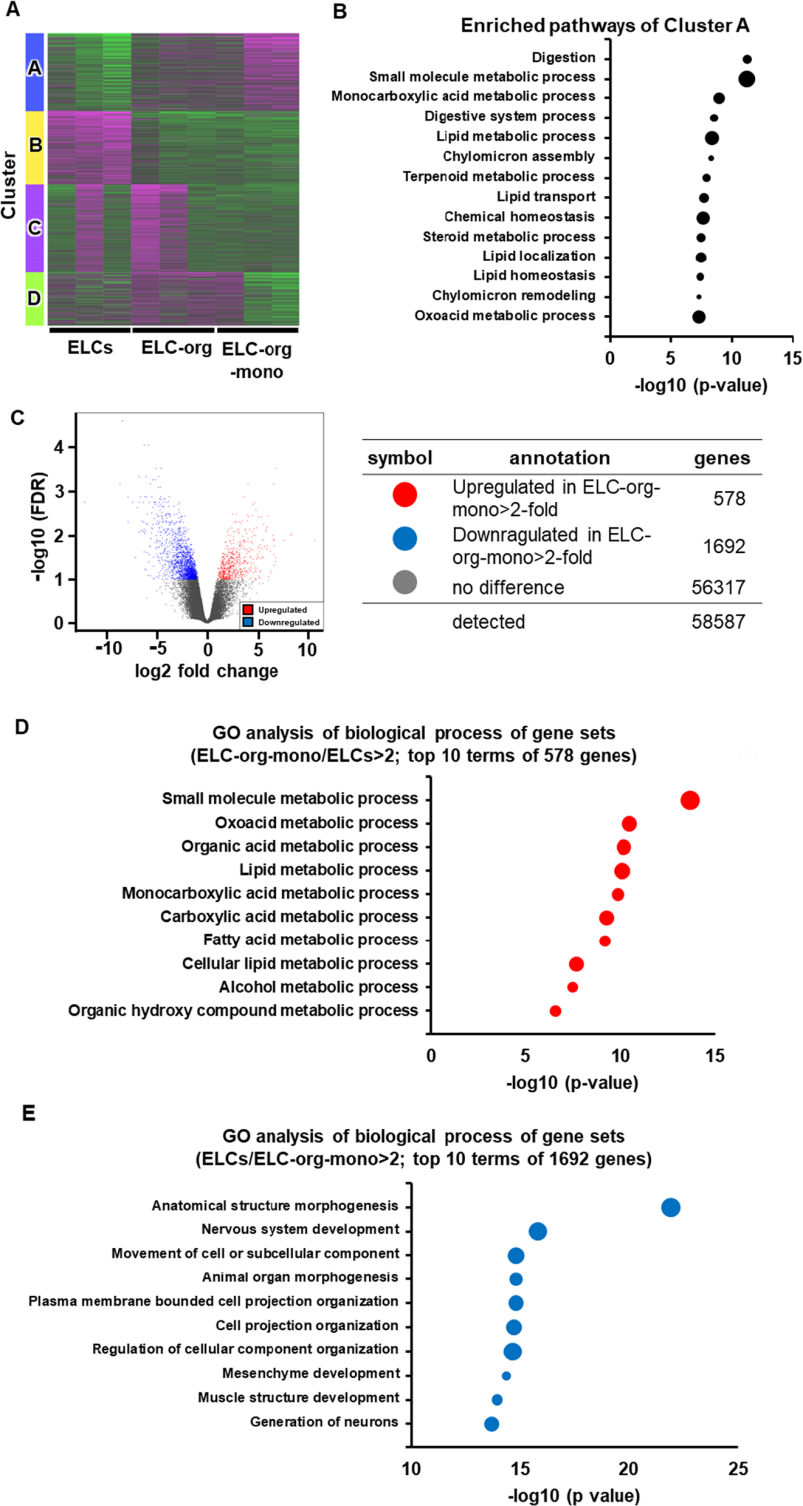
Comprehensive gene expression analysis in ELCs, ELC-org and ELC-org-mono. (**A**) The top 2000 most variable genes across all samples were visualized by heatmap and k-means clustering. Gene expression variation was calculated by Z-score, with magenta indicating an increase in expression compared to the mean across all samples, and green indicating a decrease in expression. (**B**) Enriched bubble chart of the top 14 GO pathways of enriched cluster A. The y-axis is the enriched pathway, and the x-axis is the −log 10 adjusted *P*-value. The bubble size represents the gene number. (**C**) A volcano plot comparing gene expression levels in the ELC-org-mono with those in the ELCs. (**D**) Enriched bubble chart of the top 10 up-regulated GO pathways of DEGs between the ELCs vs ELC-org-mono groups. The y-axis is the enriched pathway, and the x-axis is −log 10 adjusted *P*-value. The bubble size represents the gene number. (**E**) Enriched bubble chart of the top 10 down-regulated GO pathways of DEGs between the ELCs vs ELC-org-mono groups. The y-axis is the enriched pathway, and the x-axis is −log 10 adjusted *P*-value. The bubble size represents the gen number

Finally, to make a more detailed comparison among ELCs, ELC-org and ELC-org-mono, RNA-seq analysis was performed using next-generation sequencing (Fig. 6). K-means clustering divided the 2000 genes that were most variable among each sample into four clusters (Fig. 6A). GO enrichment analysis was performed for cluster A, which contained a group of genes whose expression was up-regulated from ELCs through ELC-org to ELC-org-mono, and the 14 terms with the smallest *P*-values were listed (Fig. 6B). In cluster A, terms related to digestion (“digestion” and “Digestive system process”) and metabolic processes (“Small molecule metabolic process”, “Monocarboxylic acid metabolic process”, “Lipid metabolic process”, “Terpenoid metabolic process”, “Steroid metabolic process” and “Oxoacid metabolic process”) were identified. These suggest that the process of producing ELC-org from ELCs and ELC-org-mono from ELC-org improved its function as a small intestine.

To examine in detail how the gene expression in the final ELC-org-mono was changed from that in the original ELCs, GO enrichment analysis was performed for the identified differentially expressed genes (DEGs). The genes with large expression changes between ELCs and ELC-org-mono were identified and shown in a volcano plot (Fig. 6C). It can be seen that 578 genes were up-regulated in ELC-org-mono from ELCs (up-regulated genes) and 1692 genes down-regulated in ELC-org-mono from ELCs (down-regulated genes). For both up-regulated genes and down-regulated genes, the 10 terms with the smallest *P*-values were listed (Fig. 6D and E). For the up-regulated genes, these top 10 terms were all related metabolic processes (“Small molecule metabolic process”, “Oxoacid metabolic process”, “Organic acid metabolic process”, “Lipid metabolic process”, “Monocarboxylic acid metabolic process”, “Carboxylic acid metabolic process”, “Fatty acid metabolic process”, “Cellular lipid metabolic process”, “Alcohol metabolic process”, and “Organic hydroxy compound metabolic process”). This suggests that metabolic activity was indeed enhanced in ELC-org-mono compared to ELCs. In the down-regulated genes, terms related mesenchymal tissue (“Nervous system development”, “Mesenchyme development,” “Muscle structure development” and “Generation of neurons”) were identified. This suggests that ELC-org-mono were able to exclude mesenchymal cells mixed with ELCs.

These results of RNA-seq showed that unlike ELCs, ELC-org-mono are composed of highly functional epithelial cells without mesenchymal cells.

## Discussion

The purpose of the present study was to establish intestinal organoids and their monolayers from human iPS cell-derived ELCs and to develop a system for stably supplying highly functional small intestinal epithelial cells for pharmaceutical research. We have succeeded in establishing mesenchymal cell-free long-term-cultured intestinal organoids from ELCs and have developed culture methods for the preparation of the highly functional monolayers from the organoids. The monolayers produced from the organoids showed a gene expression profile more similar to that in the adult human small intestine than to those in the original ELCs and their organoids. In addition, they had drug metabolism capacities comparable to those in primary cryopreserved human small intestinal cells (just after thawing) and drug transport capacities suitable for drug discovery research. Furthermore, the intestinal gene expressions were stable even after long-term culture of more than a year, and cryopreserved cells were successfully cultured directly on the cell culture inserts.

In our previous report [20], we established intestinal organoids from human iPS cells, but their function as small intestines was much lower than that of the ELC-org generated in this study. In the protocol at that time, organoid culture was started when human iPS cells were differentiated into hindgut endoderm cells (7 days differentiation), which was based on the report of McCracken et al. [35]. In this study, organoid culture was started when human iPS cells were fully differentiated into ELCs (27 days differentiation), and the culture medium for both organoids and monolayers was optimized. Thus, the period to start organoid culture, i.e., the characteristics of the cells at the start of organoid culture, is critical for the generation of highly functional intestinal organoids and their monolayers.

In this study, ELC-org-mono were used for different culture periods depending on the type of assay. Based on the results of our study of the culture period (Additional file 1: Fig. S5), we used ELC-org-mono on day 7 when examining drug metabolism activity and ELC-org-mono on day 3 when examining drug transport activity. However, ELC-org-mono demonstrated sufficient CYP3A4 and P-gp activities at 3–7 days. Therefore, ELC-org-mono can be applied for various pharmacokinetic studies anytime within the range of days 3–7.

The ELC-org produced in this study are superior to the human iPS cell-derived intestinal organoids previously reported by other groups [10–12, 29, 32, 36] in several respects. First, no special tools are required to produce ELC-org. In previous reports, organoids were generated using special plates with small irregularities that facilitate the formation of organoids [10, 11]. On the other hand, the ELC-org in this study were generated by a simple method in which ELCs are fragmented by pipetting and embedded in Matrigel. Furthermore, ELC-org do not require sorting of intestinal epithelial cells when producing monolayers. It is known that human iPS cell-derived intestinal organoids contain mesenchymal cells [29, 36]. Following this revelation, several reports required that mesenchymal cells be removed by sorting in order to produce a monolayer of highly functional intestinal epithelial cells [12, 32]. In another previous report, mesenchymal cell-free intestinal organoids were produced by sorting intestinal epithelial cells during the generation of intestinal organoids from intestinal progenitor cells [37]. Since the ELC-org in this study were composed solely of intestinal epithelial cells, functional monolayers could be formed without sorting, excluding mesenchymal cells. This requirement of only a simple procedure to prepare for the intestinal monolayers constitutes an advantage for the application to pharmaceutical research.

In FACS analysis, the EpCAM-positive cell rate was 100% not only in ELC-org (Fig. 3C) but also in pre-ELC-org (Additional file 1: Fig. S3). For reference, the EpCAM-positive cell rate was 56% in intestinal organoids generated from human iPS cell-derived hindgut endoderm cells, which were differentiated for a week, in our previous report [20]. The differentiation period for the generation of pre-ELC-org in our present study was 10 days longer than that in the previous report. Thus at least 17 days would be required, in addition to the optimized differentiation protocol, to differentiate small intestinal epithelial cells without mesenchyme from human iPS cells. On the other hand, the Villin-positive cell rate in ELC-org-mono was decreased from that in ELC-org **(**Fig. 3C**)**. This trend was confirmed in a previous report on human duodenal-derived organoids [20]. It is likely that the monolayer culture promoted the differentiation of intestinal cells and generated a variety of differentiated cells, resulting in a decrease in the number of absorptive epithelial (Villin-positive). In fact, differentiated cells, such as endocrine, Paneth, and goblet cells, were observed in ELC-org-mono (Fig. 3F).

In this study, we also determined the optimum medium to generate highly functional monolayers (ELC-org-mono) from the ELC-org. By replacing the organoid culture medium with the intestinal maturation medium, which is identical to the final medium used to generate ELCs from human iPS cells, the ELC-org-mono regained extremely high activity of the most important drug-metabolizing enzyme, CYP3A4 (Figs. 1C and 2A). We also found that ELC-org-mono could be made more functional by modifying the composition of the intestinal maturation medium (Additional file 1: Fig. S8). This was especially the case when EGF was excluded from intestinal maturation medium, resulting in an additional augmentation of CYP3A4 activity, without any significant change in P-gp activity (Additional file 1: Fig. S8C and S8D). Further improvement of the intestinal functions in ELC-org-mono might be possible by conducting detailed screening and optimization of the medium composition.

The P-gp activity in ELC-org-mono had a higher efflux ratio (Fig. 4C) than that in previous reports [32, 38, 39] and was comparable to that of human duodenal organoid-derived monolayers and Caco-2 cells [17]. Guidance from the U.S. Food and Drug Administration (FDA) requires that drug candidate compounds be evaluated as substrates for MDR1 (P-gp) and BCRP in the early stages of drug development (https://www.fda.gov/drugs/guidance-compliance-regulatory-information/guidances-drugs). ELC-org-mono have both P-gp and BCRP activities and are useful evaluation systems that satisfy these requirements.

## Conclusions

In the present study, we have succeeded in developing a highly functional monolayer platform (ELC-org-mono) from human iPS cell-derived ELCs through an organoid culture and have demonstrated its suitability for pharmacokinetic studies. ELC-org-mono were free of the contamination of mesenchymal cells and composed of the intestinal differentiated cells identified in the human small intestine. Their gene expression levels of many pharmacokinetic enzymes and transporters were comparable to those in the human small intestine. Furthermore, they had high drug metabolism and drug transport activities that could be applied to pharmacokinetic studies. It is quite interesting and important that the drug-metabolizing capacities in ELC-org-mono mimic those in primary cryopreserved human small intestinal cells (just after thawing). This functionality was still present in ELC-org-mono, which were generated from organoids cultured for more than a year. The present method together with ELC-org and ELC-org-mono will contribute not only to a dramatically improvement in the drug discovery process, but also to a variety of basic studies and regenerative-medicine studies using human intestinal cells.

### Supplementary Information

**Additional file 1: **Supplementary Materials and Methods.

## Data Availability

All the RNA-seq data in this work have been deposited at GEO (https://www.ncbi.nlm.nih.gov/geo/) under accession number GSE240322.
