# Supplementary material for: Functional intestinal monolayers from organoids derived from human iPS cells for drug discovery research
Source: Stem Cell Res Ther. 2024 Feb 29;15:57. doi: 10.1186/s13287-024-03685-5 (PMC10905936; doi:10.1186/s13287-024-03685-5)
Supplement: Supplementary file 1 — Additional file 1: Supplementary Materials and Methods. [file 13287_2024_3685_MOESM1_ESM.docx]

Supplementary Materials and Methods

**Proliferative capacity**

The number of living cells was evaluated by quantifying intracellular ATP using the CellTiter-Glo 3D Cell Viability Assay (Promega) according to the manufacturer’s instruction. The luminescence intensity was measured with a multimode microplate reader (Berthold Technologies) for 0.25 seconds per well.

**Cryopreservation of ELC-org**

ELC-org were dissociated into single cells according to our previous report^1^. Singe cells were resuspended in STEM-CELLBANKER (ZENOGEN PHARMA), frozen overnight at -80°C, and then frozen at -150°C for 2 weeks.

**ELC-org-mono prepared from cryopreserved cell suspensions**

The cryopreserved ELC-org cell suspensions were thawed, seeded directly onto Matrigel-coated cell culture inserts (24-well plate, 0.4 μm pore size, PET Membrane, Corning) or 96 well plates (Thermo Fisher Scientific) to a density of 5.0×10^5^ cells/well. The monolayers were cultured for 3 to 7 days with intestinal maturation medium.

## **CYP3A4 Induction**

ELC-org-mono were cultured in intestinal maturation medium (IMM) without VD3 (-V) for 5 days. Then, they were treated with/without 100 nM VD3 or 20 mM rifampicin (RIF; FUJIFILM Wako) for last 2 days; these agents are known to induce CYP3A4^2,3^.

## **Modification of intestinal maturation medium**

ELC-org-mono were cultured in intestinal maturation medium (IMM) for 1 day. For the next 6 days, they were cultured in IMM or IMM without EGF (-E), SB (-SB), LY (-LY), VD3 (-V) or PD (-PD).

## **References**

1. Yamashita, T. *et al.* Monolayer platform using human biopsy-derived duodenal organoids for pharmaceutical research. *Mol. Ther. - Methods Clin. Dev.* **22**, 263–278 (2021).

2. Thummel, K. E. *et al.* Transcriptional Control of Intestinal Cytochrome P-4503A by 1α,25-Dihydroxy Vitamin D3. *Mol. Pharmacol.* **60**, 1399–1406 (2001).

3. Glaeser, H., Drescher, S., Eichelbaum, M. & Fromm, M. F. Influence of rifampicin on the expression and function of human intestinal cytochrome P450 enzymes. *Br. J. Clin. Pharmacol.* **59**, 199 (2005).

Supplementary Figure

**Figure S1**


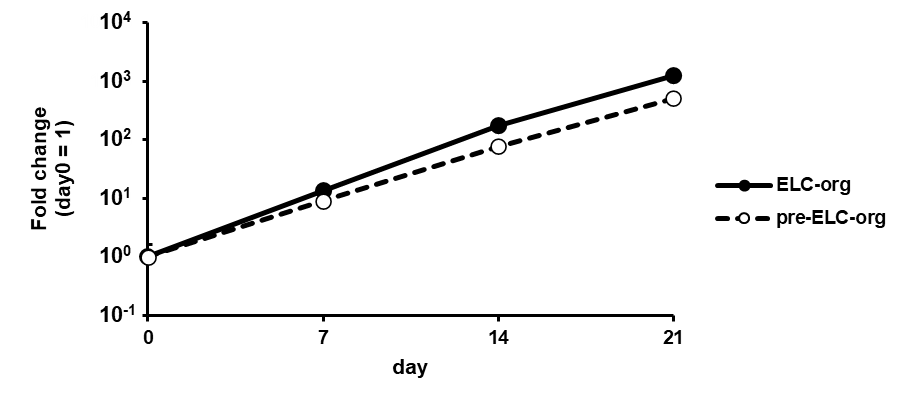


**Figure S1. ELC-org** **had higher proliferative capacity than pre-ELC-org.**

The growth curves of ELC-org and pre-ELC-org are shown. Cell growth was measured by the CellTiter-Glo 3D Cell Viability Assay. Data acquisitions of each organoid were performed every 7 days (until day 21) after passage. Data are presented as means ± S.D. (n = 3, biological replicate).

**Figure S2**


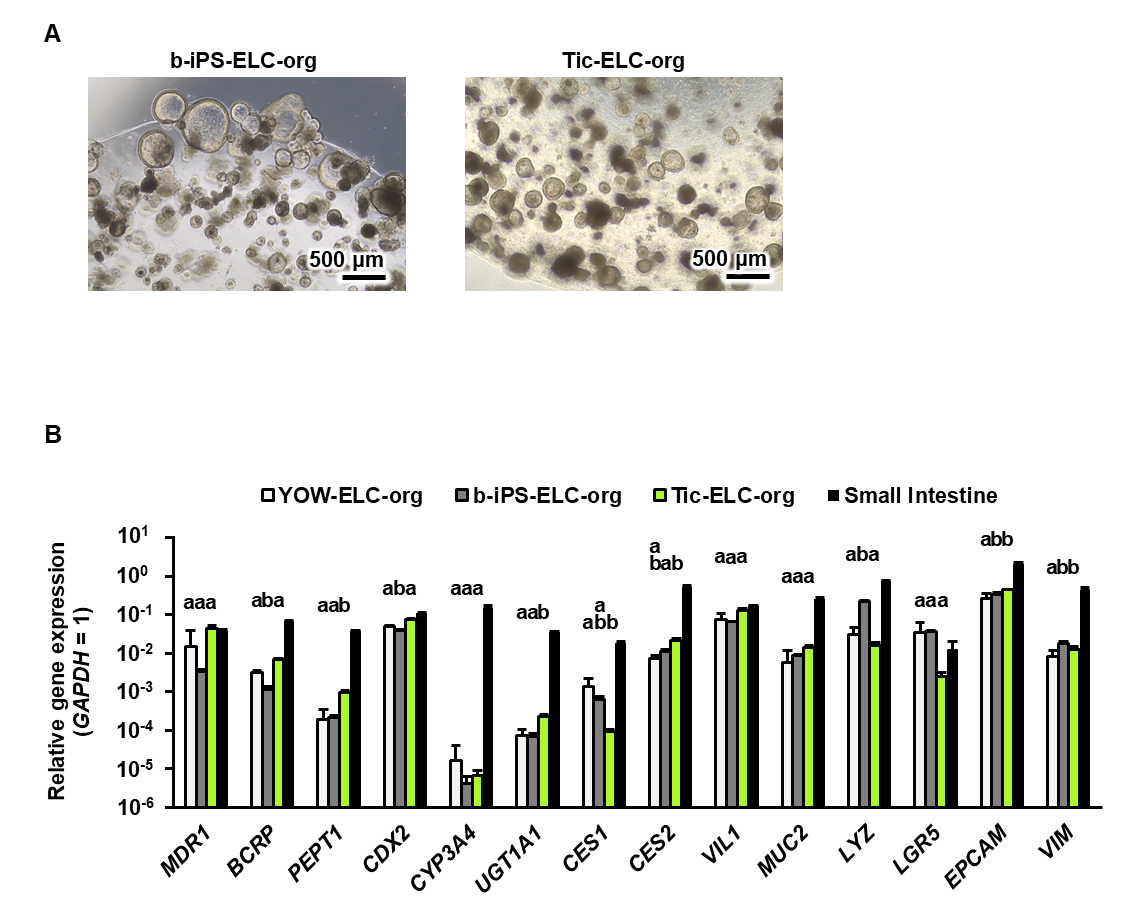


**Figure S2. ELC-org were generated from other iPS cell lines.**

(A) Phase-contrast images of ELC-org generated from other iPS cell lines (b-iPS and Tic) are shown. (B) The gene expression levels of drug transporters (*MDR1*, *BCRP*, *PEPT1*), drug-metabolizing enzymes (*CYP3A4*, *UGT1A1*, *CES1*, *CES2*), intestinal cell markers (*CDX2*, *VIL*, *MUC2*, *LYZ*, *LGR5*, *EPCAM*) and a mesenchymal cell marker (*VIM*) were examined in ELC-org generated from various iPS cell lines (YOW, b-iPS and Tic) and the human small intestine by qRT-PCR. The *GAPDH* expression level was taken as 1.0. All data represent the mean ± S.D. (*n* = 3, biological replicates). Statistical significance was evaluated by one-way ANOVA followed by Tukey’s post hoc test. Groups that do not share the same letter had significantly different results (*p* < 0.05).

**Figure S3**

**
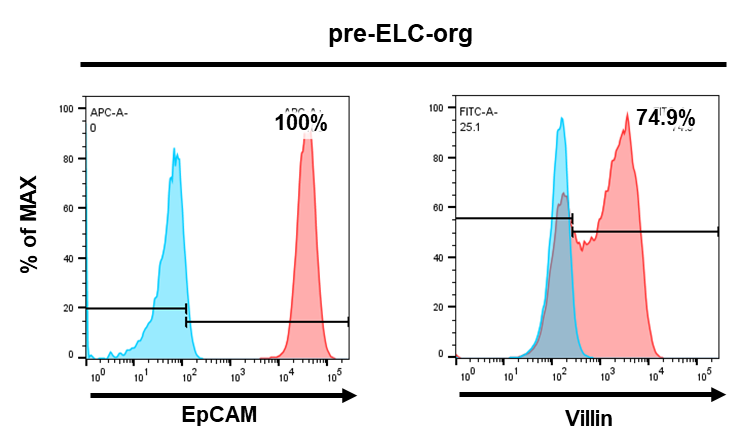
**

**Figure S3. Percentages of EpCAM- and Villin-positive cells in pre-ELC-org.**

Percentages of EpCAM- and Villin-positive cells in pre-ELC-org were measured by FACS analysis. Negative control (blue) and stained cells (red) are represented.

**Figure S4**

**
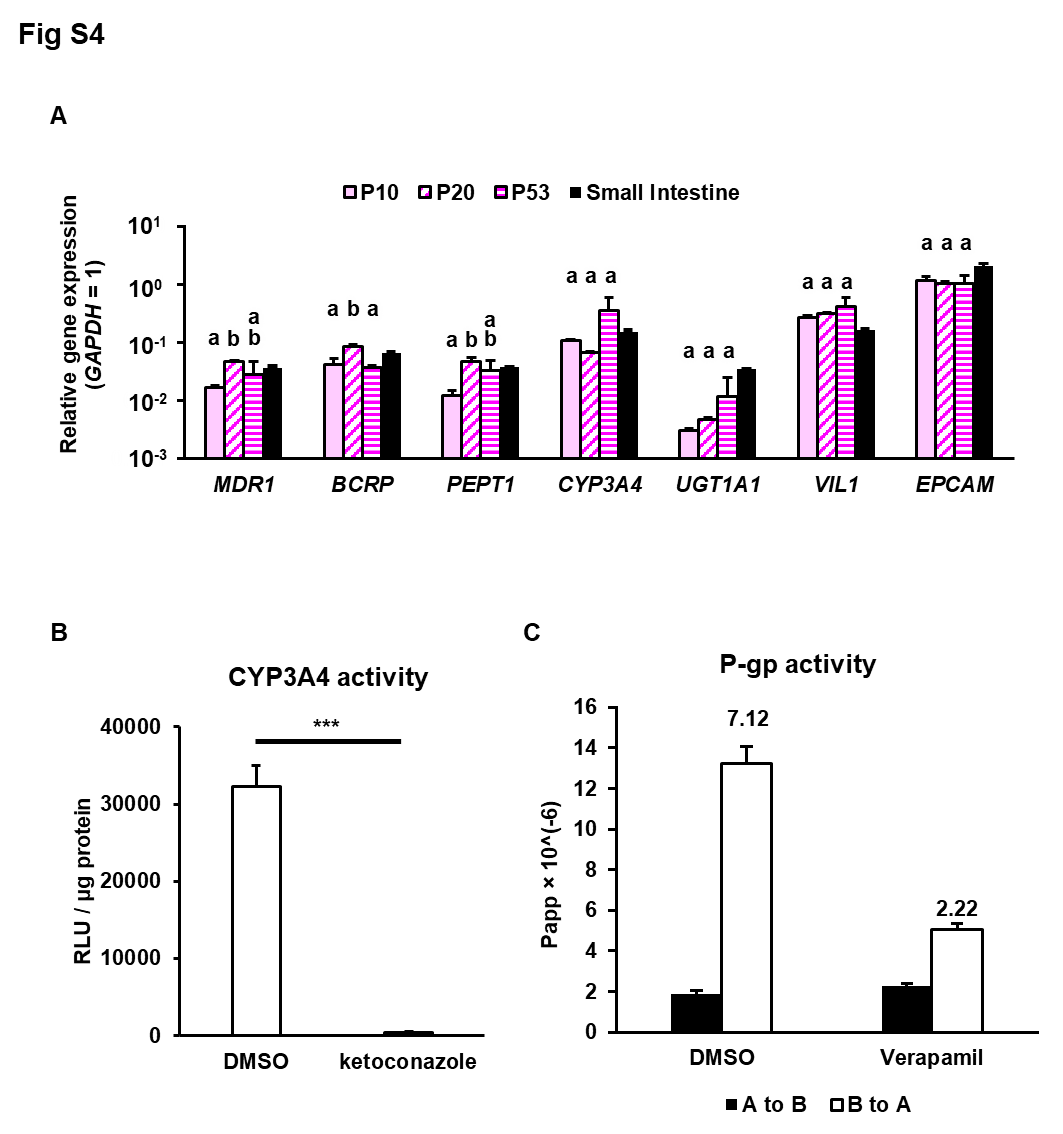
**

**Figure S4. ELC-org maintained their function even after long-term culture**

(A) The gene expression levels of drug transporters (*MDR1*, *BCRP*, *PEPT1*), drug-metabolizing enzymes (*CYP3A4*, *UGT1A1*), and intestinal cell markers (*VIL*, *EPCAM*) were examined by qRT-PCR in each monolayer derived from ELC-org with 10, 20, and 53 successive passages and in the human small intestine. The *GAPDH* expression level was taken as 1.0. Statistical significance was evaluated by one-way ANOVA followed by Tukey’s post hoc test. Groups that do not share the same letter had significantly different results (p < 0.05). (B) The CYP3A4 activity in the monolayer derived from ELC-org with 51 successive passages was examined by using the P450-Glo CYP3A4 assay kit in the presence or absence of 10 µM ketoconazole (a CYP3A4 inhibitor). Statistical analyses were performed using the unpaired two-tailed Student’s t-test (****p* < 0.005). (C) The permeabilities of Rhodamine123 (a P-gp substrate) in the monolayer derived from ELC-org with 52 successive passages were measured. The efflux ratio (P_app B to A_/P_app A to B_) of each group is shown above the bar. All data represent the mean ± S.D. (*n* = 3, biological replicates).

**Figure S5**

**
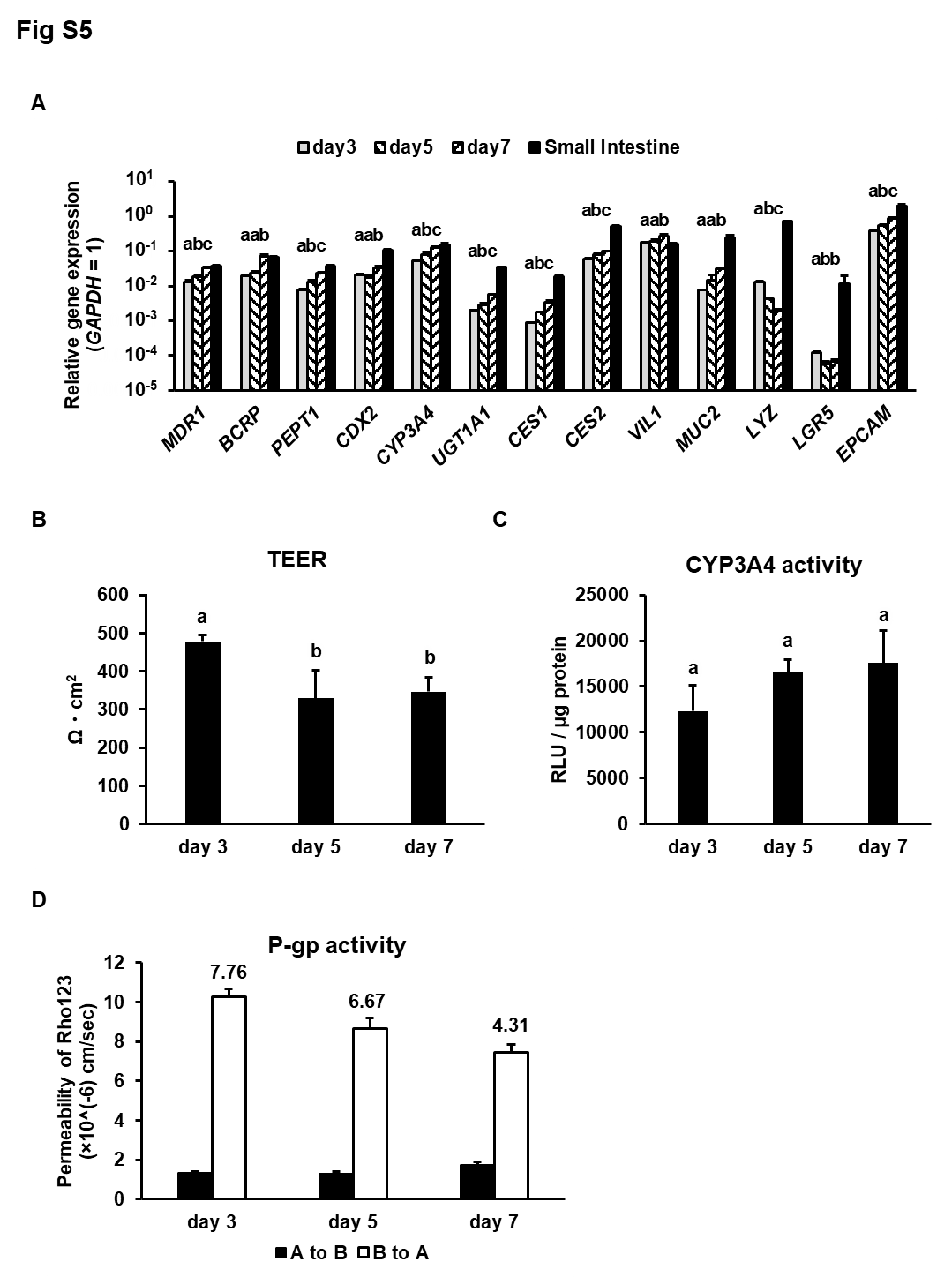
**

**Figure S5. Optimal monolayer culture period for ELC-org-mono**

(A) The gene expression levels of drug transporters (*MDR1*, *BCRP*, *PEPT1*), drug-metabolizing enzymes (*CYP3A4*, *UGT1A1*, *CES1*, *CES2*) and intestinal cell markers (*CDX2*, *VIL*, *MUC2*, *LYZ*, *LGR5*, *EPCAM*) were examined by qRT-PCR in ELC-org-mono cultured for different culture periods and in the human small intestine. The *GAPDH* expression level was taken as 1.0. (B) The TEER values in each cell monolayer were measured by Millicell-ERS2. (C) The CYP3A4 activity in each cell monolayer was examined by using the P450-Glo CYP3A4 assay kit in the presence or absence of 10 µM ketoconazole (a CYP3A4 inhibitor). (D) The permeabilities of Rhodamine123 (a P-gp substrate) in each cell monolayer were measured. The efflux ratio (P_app B to A_/P_app A to B_) of each group is shown above the bar. All data represent the mean ± S.D. (*n* = 3, biological replicates). Statistical significance was evaluated by one-way ANOVA followed by Tukey’s post hoc test. Groups that do not share the same letter had significantly different results (*p* < 0.05).

**Figure S6**

**
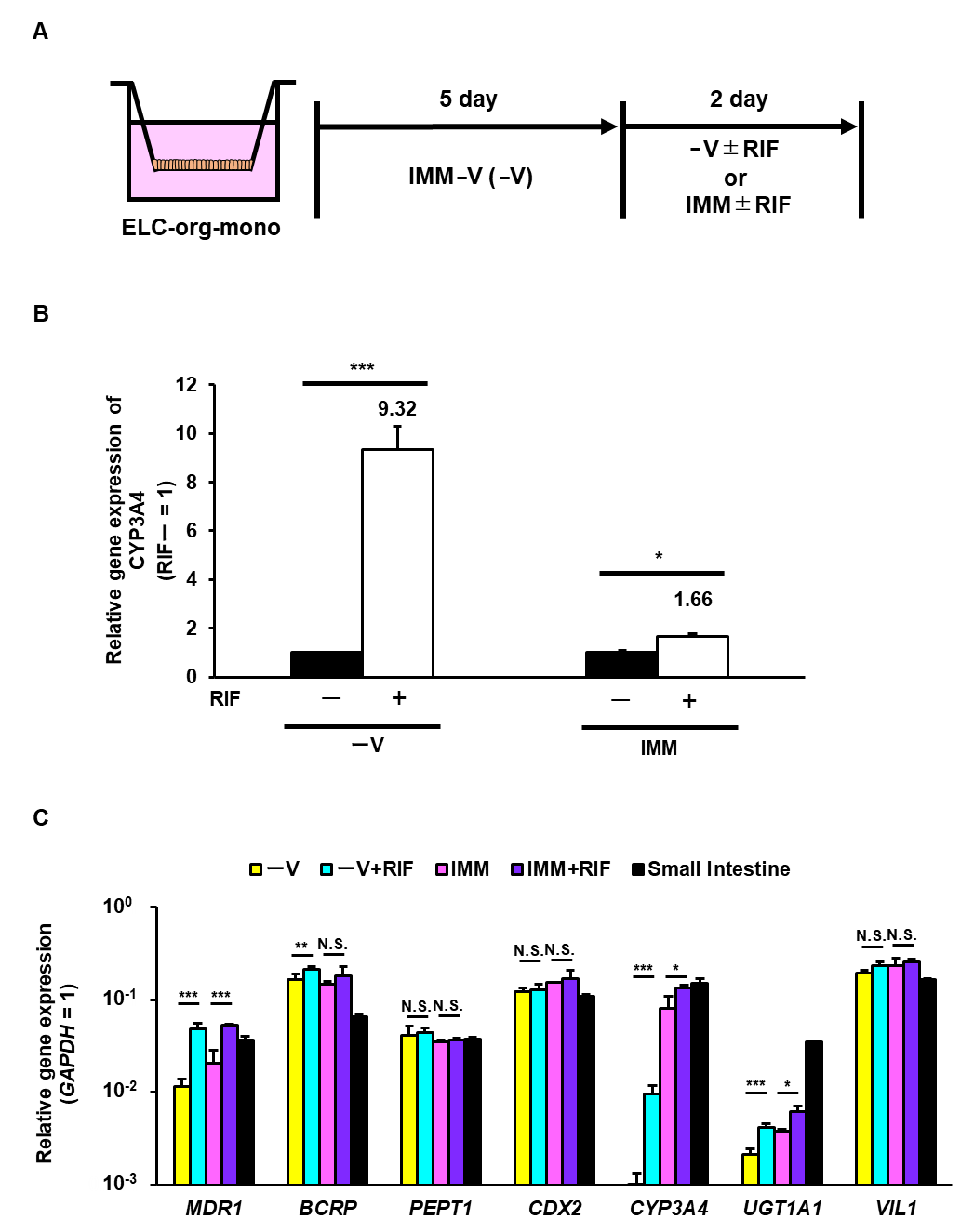
**

**Figure S6. CYP3A4 induction in ELC-org-mono**

ELC-org-mono were cultured with intestinal maturation medium with/without VD3 or RIF (-V or IMM, respectively). (A) Diagram of the protocol for CYP3A4 induction. (B) The gene expression level of *CYP3A4* was examined in ELC-org-mono with or without RIF by qRT-PCR. The expression level in ELC-org-mono without RIF was taken as 1.0. Statistical analyses were performed using the unpaired two-tailed Student’s t-test (**p* < 0.05, ****p* < 0.005). (C) The gene expression levels of drug transporters (*MDR1*, *BCRP*, *PEPT1*), drug-metabolizing enzymes (*CYP3A4*, *UGT1A1*) and intestinal cell markers (*CDX2*, *VIL*) were examined in ELC-org-mono with or without RIF and the human small intestine by qRT-PCR. The *GAPDH* expression level was taken as 1.0. Groups that do not share the same letter had significantly different results (*p* < 0.05). All data represent the mean ± S.D. (*n* = 3, biological replicates). Statistical analyses were performed using the unpaired two-tailed Student’s t-test (**p* < 0.05, ***p* < 0.01, ****p* < 0.005). N.S. means “Not Significant”.

**Figure S7**

**
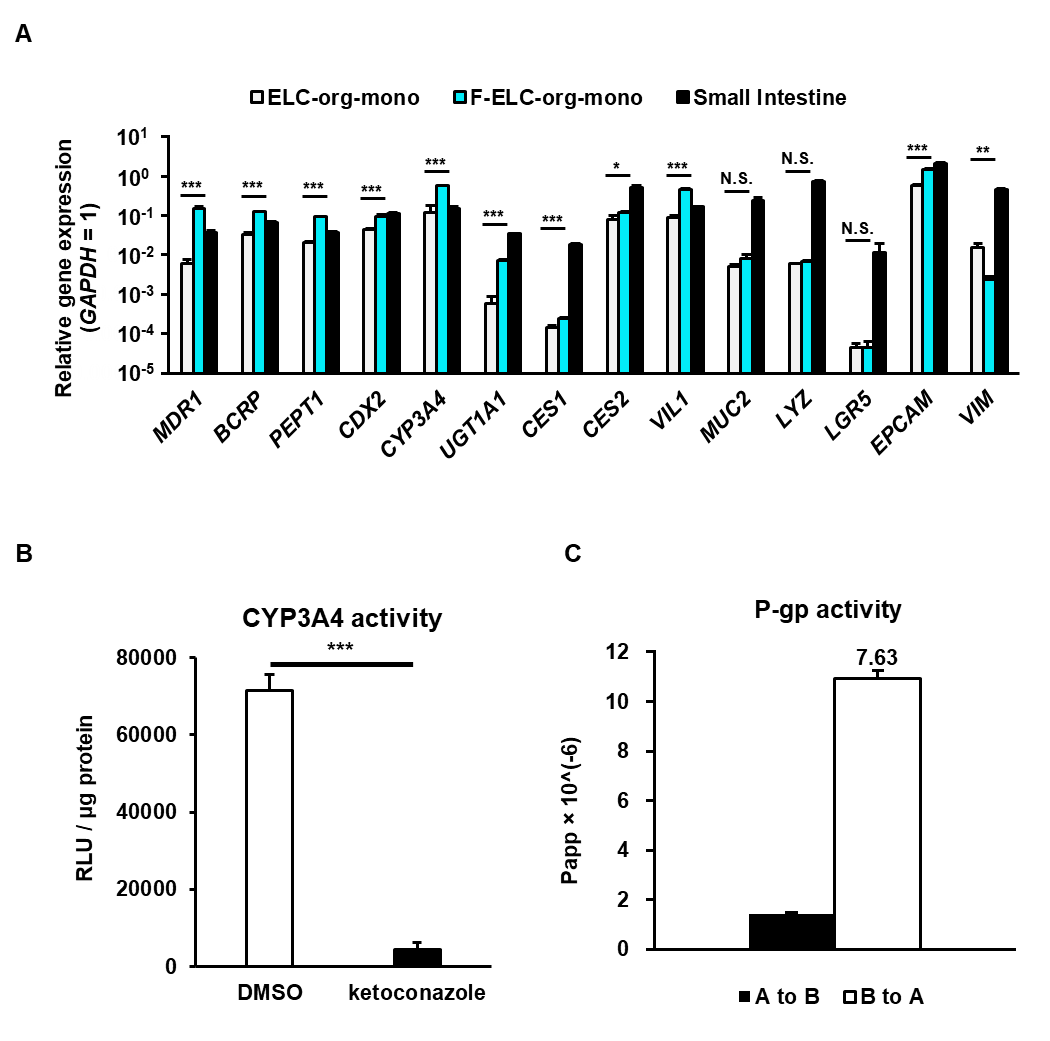
**

**Figure S7. ELC-org can form functional monolayers from cryopreserved cell suspensions**

ELC-org were dissociated into single cells and cryopreserved for 2 weeks. Then, cryopreserved ELC-org cell suspensions were thawed, seeded directly onto cell culture inserts, and cultured for 3 to 7 days (F-ELC-org-mono). (A) The gene expression levels of drug transporters (*MDR1*, *BCRP*, *PEPT1*), drug-metabolizing enzymes (*CYP3A4*, *UGT1A1*, *CES1*, *CES2*), intestinal cell markers (*CDX2*, *VIL*, *MUC2*, *LYZ*, *LGR5*, *EPCAM*) and a mesenchymal cell marker (*VIM*) were examined in F-ELC-org-mono by qRT-PCR. As a control, the gene expression levels in ELC-org-mono without cryopreservation and in the human small intestine were examined. The *GAPDH* expression level was taken as 1.0. Statistical analyses were performed using the unpaired two-tailed Student’s t-test (**p* < 0.05, ***p* < 0.01, ****p* < 0.005). N.S. means “Not Significant”. (B) The CYP3A4 activity in F-ELC-org-mono was examined by using the P450-Glo CYP3A4 assay kit in the presence or absence of 10 µM ketoconazole (a CYP3A4 inhibitor). Statistical analyses were performed using the unpaired two-tailed Student’s t-test (****p* < 0.005). (C) The permeabilities of Rhodamine123 (substrate for P-gp) in F-ELC-org-mono were measured. The efflux ratio (P_app B to A_/P_app A to B_) of each group is shown above the bar. All data represent the mean ± S.D. (*n* = 3, biological replicates).

**Figure S8**


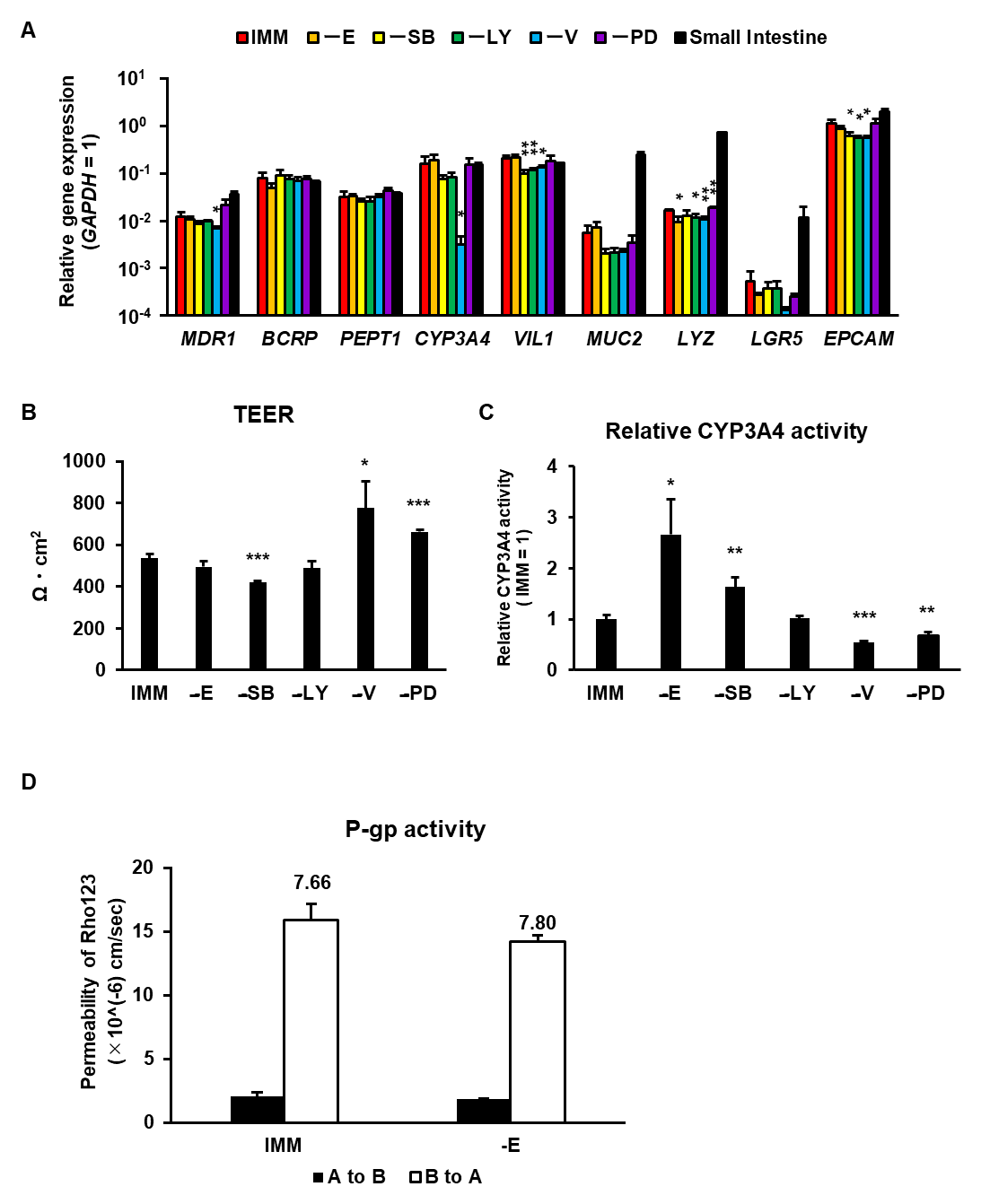


**Figure S8.**  **Modification of the medium composition makes ELC-org-mono more functional**

ELC-org-mono were cultured in IMM or IMM without EGF (-E), SB (-SB), LY (-LY), VD3 (-V) or PD (-PD). (A) The gene expression levels of drug transporters (*MDR1*, *BCRP*, *PEPT1*), drug-metabolizing enzymes (*CYP3A4*, *UGT1A1*, *CES1*, *CES2*) and intestinal cell markers (*CDX2*, *VIL*, *MUC2*, *LYZ*, *LGR5*, *EPCAM*) were examined. As a control, the gene expression levels in the human small intestine were examined. The *GAPDH* expression level was taken as 1.0. (B) The TEER values in each cell monolayer were measured by Millicell-ERS2. (C) The CYP3A4 activity was examined by using the P450-Glo CYP3A4 assay kit. (D) The permeabilities of Rhodamine123 (a P-gp substrate) in each cell monolayer were measured. The efflux ratio (P_app B to A_/P_app A to B_) of each group is shown above the bar. All data represent the mean ± S.D. (*n* = 3, biological replicates). Statistical analyses were performed using the unpaired two-tailed Student’s t-test. * indicates significant difference in the quantitative value compared with ELC-org-mono cultured in IMM (**p* < 0.05, ***p* < 0.01, ****p* < 0.005).

**Table S1. Medium composition of intestinal maturation medium**

| **Name** | **final conc.** |
| --- | --- |
| Dulbecco’s Modified Eagle’s Medium（High Glucose） | (Basal medium) |
| GlutaMAX | 1 mM |
| penicillin/streptomycin | 1% |
| MEM NEAA | 1% |
| B-27 Supplement | 0.5× |
| KnockOut Serum Replacement | 10% |
| EGF | 50 ng/ml |
| SB431542 | 2 µM |
| 1α,25-dihydroxyvitamin D3 | 100 nM |
| LY2090314 | 3 nM |
| PD0325901 | 3 µM |

**Table S2. Primers used in the real-time RT-PCR analysis**

| **Gene Symbol** | **Primers (forward/reverse; 5’ to 3’)** |
| --- | --- |
| *BCRP* | TGCAACATGTACTGGCGAAGA/TCTTCCACAAGCCCCAGG |
| *CDX2* | TCCGTGTACACCACTCGATATT/GGAACCTGTGCGAGTGGAT |
| *CES1* | ACCCCTGAGGTTTACTCCACC/TGCACATAGGAGGGTACGAGG |
| *CES2* | CTAGGTCCGCTGCGATTTG/TGAGGTCCTGTAGACACATGG |
| *CYP3A4* | AAGTCGCCTCGAAGATACACA/AAGGAGAGAACACTGCTCGTG |
| *EPCAM* | AATCGTCAATGCCAGTGTACTT/TCTCATCGCAGTCAGGATCATAA |
| *GAPDH* | GGTGGTCTCCTCTGACTTCAACA/GTGGTCGTTGAGGGCAATG |
| *LGR5* | CTCCCAGGTCTGGTGTGTTG/GAGGTCTAGGTAGGAGGTGAAG |
| *LYZ* | GGCCAAATGGGAGAGTGGTTA/CCAGTAGCGGCTATTGATCTGAA |
| *MDR1* | GCCAAAGCCAAAATATCAGC/TTCCAATGTGTTCGGCATTA |
| *MUC2* | GAGGGCAGAACCCGAAACC/GGCGAAGTTGTAGTCGCAGAG |
| *PEPT1* | AATGTTCTGGGCCTTGTTTG/CATCTGATCGGGCTGAATTT |
| *UGT1A1* | CTGTCTCTGCCCACTGTATTCT/TCTGTGAAAAGGCAATGAGCAT |
| *VIL1* | AGCCAGATCACTGCTGAGGT/TGGACAGGTGTTCCTCCTTC |
| *VIM* | AGTCCACTGAGTACCGGAGAC/CATTTCACGCATCTGGCGTTC |

**Table S3. The primary antibodies used for immunocytochemistry**

| **Name** | **Host** | **Company** | **Catalog number** |
| --- | --- | --- | --- |
| Anti-Cytochrome P450 3A4 antibody | rabbit | abcam | ab135813 |
| Anti-Villin antibody | rabbit | abcam | ab130751 |
| Anti-E Cadherin antibody | rabbit | abcam | ab40772 |
| Anti-CDX2 antibody | rabbit | abcam | ab76541 |
| Anti-Chromogranin A antibody | mouse | abcam | ab715 |
| Anti-Lysozyme antibody | mouse | abcam | ab36362 |
| Anti-MUC2 antibody | mouse | abcam | ab118964 |
| Villin Antibody | mouse | Santa Cruz Biotechnology | sc-58897 |
| ZO-1 Polyclonal Antibody | rabbit | Thermo Fisher Scientific | 40-2200 |

**Table S4. The secondary antibodies used for immunocytochemistry**

| **Name** | **Host** | **Company** | **Catalog number** |
| --- | --- | --- | --- |
| Donkey anti-mouse IgG Secondary Antibody,  Alexa Fluor 594 conjugate | donkey | Thermo Fisher Scientific | A-21203 |
| Donkey anti-rabbit IgG Secondary Antibody,  Alexa Fluor 488 conjugate | donkey | Thermo Fisher Scientific | A-21206 |

**Table S5. Antibodies used in FACS analysis**

| **Name** | **Company** | **Catalogue No.** | **Dilution** |
| --- | --- | --- | --- |
| CD326 (EpCAM)-APC,  human monoclonal | Miltenyi Biotec | 130-091-254 | 1:50 |
| Anti-Villin antibody [SP145] | abcam | ab130751 | 1:50 |
| Isotype Control Antibody, mouse IgG1, APC | Miltenyi Biotec | 130-113-196 | 1:50 |
| Donkey anti-rabbit IgG Secondary Antibody,  Alexa Fluor 488 conjugate | Thermo Fisher Scientific | A-21206 | 1:1000 |

**Table S6. Substrates used in UPLC-MS/MS analysis**

| **Substrate** | **Final conc. (**μM**)** | **Metabolite** | **Corresponding　CYPs** |
| --- | --- | --- | --- |
| Midazolam | 0.1 | 1-hydroxy midazolam (MDOH) | CYP3A4 |
| Testosterone | 1 | 6β-hydroxy testosterone (TSOH) | CYP3A4 |
| (±)-Bufuralol hydrochloride | 0.1 | 1-hydroxy bufuralol (BFOH) | CYP2D6 |
| Diclofenac sodium salt | 0.1 | 4-hydroxydiclofenac (DFOH) | CYP2C9 |
| (S)-(+)-Mephenytoin | 8 | 4-hydroxymephenytoin (MPOH) | CYP2C19 |
